# Characterizing Sex Differences in Mitochondrial Dysfunction After Severe Traumatic Brain Injury in Mice

**DOI:** 10.1089/neur.2023.0046

**Published:** 2023-09-25

**Authors:** Olivia J. Kalimon, Hemendra J. Vekaria, Gopal V. Velmurugan, W. Brad Hubbard, Patrick G. Sullivan

**Affiliations:** ^1^ Department of Neuroscience, University of Kentucky, Lexington, Kentucky, USA.; ^2^ Spinal Cord and Brain Injury Research Center, University of Kentucky, Lexington, Kentucky, USA.; ^3^ Lexington VA Healthcare System, Lexington, Kentucky, USA.; ^4^ Department of Physiology, University of Kentucky, Lexington, Kentucky, USA.

**Keywords:** calcium loading, CNS injury, mitochondrial bioenergetics, time course

## Abstract

Traumatic brain injury (TBI) is caused by an impact or penetrating injury to the head resulting in abnormal brain function. Mitochondrial dysfunction is an important hallmark of TBI and has been thoroughly studied in male rodent models of brain injury, but relatively little is known about these outcomes in females. These studies were designed to examine sex as a biological variable for mitochondria-related outcomes after the severe controlled cortical impact (CCI) mouse model of TBI. Synaptic and non-synaptic mitochondria were isolated from the sham- or CCI-injured cortex as well as the hippocampus ipsilateral to the craniotomy 3, 12, 24, or 48 h post-surgery, and then bioenergetics were measured. Subtle variations were observed in the timeline of mitochondrial dysfunction between sexes. Non-synaptic cortical mitochondria from injured females showed early impairment at 12 h post-CCI compared to mitochondria from injured males at 24 h post-CCI. Contrastingly, in the synaptic fraction, mitochondria from injured males showed early impairment at 12 h post-CCI, whereas mitochondria from injured females showed impairment at 24 h post-CCI. Based on bioenergetic impairments at 24 h post-CCI, synaptic and non-synaptic mitochondrial calcium loading was also measured at this time point. Consistent with bioenergetic data at 24 h, non-synaptic mitochondria from injured males had increased calcium loading compared to uninjured control, but this effect was not observed in females. Finally, histological assessment of cortical tissue sparing in each sex was measured at 7 days post-injury. There was a lack of sex-based differences in cortical tissue sparing after severe CCI. Overall, there were some subtle sex differences in mitochondrial outcomes after CCI, but these findings were not statistically significant. This study highlights the importance of utilizing both sexes when measuring mitochondrial function after severe CCI.

## Introduction

Traumatic brain injury (TBI) is a serious health concern resulting from a penetrating or non-penetrating injury to the head that affects normal brain function. The most recent data from the Centers for Disease Control and Prevention reported >61,000 TBI-related deaths in the United States in 2019 and >223,000 TBI-related hospitalizations in 2018.^1,2^ Mitochondrial dysfunction is a key hallmark of TBI neuropathology and has been a target of interest to diminish secondary injury post-TBI.^3–9^ A recent review by Gupte and colleagues highlighted sex differences in both human and rodent brain injury and found that a large percentage of severe TBI studies reported improved outcomes in both women and female rodents compared to their male counterparts.^10^ The researchers emphasized that mitochondrial function may be an important source of sex differences after TBI. Most of the clinical and pre-clinical research has been performed in males, leaving a significant knowledge gap on the effects of TBI on mitochondrial dysfunction in females and thus a substantial gap of how to treat the other fraction of the clinical population.

Mitochondria are considered the “powerhouses” of cells because of their important roles in energy production, cellular homeostasis, and calcium (Ca^2+^) handling.^11^ Mitochondria are major producers of reactive oxygen/nitrogen species (ROS/RNS) as the natural by-product of their function.^11–13^ Typically, these species are scavenged by antioxidants (i.e., manganese superoxide dismutase and glutathione peroxidase) to reduce the amount of free radical damage.^14–17^ After TBI, neurons experience an increase in excitotoxic glutamate that results in an influx of Ca^2+^.^13,15,18^ Mitochondria are one of the key organelles that maintain normal intracellular Ca^2+^ levels, and they do so in a membrane potential (ΔΨ_m_)-dependent manner.^18,19^ Excessive increases in intramitochondrial Ca^2+^ have been shown to alter mitochondrial homeostasis by inhibiting adenosine triphosphate (ATP) synthesis and generating excess ROS/RNS.^8^ Eventually, mitochondrial ΔΨ_m_ becomes too low (∼–100 mV), resulting in ATP consumption and mitochondrial permeability transition, a process in which a non-specific pore opens, allowing the release of Ca^2+^ and other mitochondrial proteins into the cytosol to trigger neuronal cell death.^3,11,20–22^

Mitochondria are found in all cell types in the brain, and just as cells have different functions, the mitochondria within these cells have different profiles. Our lab has optimized a technique for isolating two distinct fractions of mitochondria within the brain so that we can examine the bioenergetic profiles from small amounts of tissue.^23^ These fractions are non-synaptic mitochondria and synaptic mitochondria, with synaptic mitochondria being our primary therapeutic target of interest.^6,24^ Non-synaptic mitochondria are glia-enriched, whereas synaptic mitochondria are primarily neuronal.^12,23–25^ Synaptic mitochondria are synthesized in the cell body of neurons and then trafficked down the axon or dendrite to the pre- and post-synaptic terminals, respectively.^19,25–28^ Mitochondrial transport is disrupted after TBI, which likely contributes to neuronal dysfunction after injury (reviewed by Shin and colleagues^29^). Mitochondria within the synapse are continuously exposed to Ca^2+^ because of the large influxes involved in synaptic transmission, making these mitochondrial fractions more sensitive to Ca^2+^ overload than non-synaptic mitochondria, even in uninjured conditions.^19^

After TBI, synaptic mitochondria experience earlier and greater bioenergetic deficits and oxidative damage compared to non-synaptic mitochondria.^12,30^ Our lab has recently shown that direct rescue of synaptic mitochondrial bioenergetics improves long-term cognitive function in a mouse model of TBI, indicating the value of studying these fractions separately.^6^ Importantly, the study by Hubbard and colleagues used both male and female mice, though those experiments were not powered to detect sex differences.^6^

A study conducted to examine sex differences in naïve mitochondria found total mitochondria from the brain of females had higher Complex I–driven respiration compared to mitochondria from male mice.^31^ Past research has shown that brain mitochondria from naïve female mice have a lower Ca^2+^ capacity compared to males, which may hint at increased resiliency of male-derived mitochondria to injury.^32,33^ It has been shown that both synaptic and non-synaptic mitochondria from the female mouse brain produce less hydrogen peroxide than that of males.^34^ There appears to be sex differences in mitochondrial function in naïve mice; however, there is little information available regarding sex differences in mitochondrial function post-TBI (reviewed by Kalimon and colleagues in 2021).^35^

A study by Greco and colleagues found that male rats had a significantly impaired respiratory control ratio, a measure of mitochondrial respiratory health, compared to their uninjured control 24 h after severe controlled cortical impact (CCI); however, female rats did not have this injury effect.^36^ The researchers also found increased mitochondrial peroxide production in males, but no change in peroxide production in females.^36^ This study suggests that female-derived mitochondria may have innate protection to CCI-induced injury, though those experiments were performed in rats. No other studies have measured sex differences in mitochondrial function in mice. To address this knowledge gap, we have designed these studies to identify sex differences in synaptic and non-synaptic mitochondrial dysfunction after the severe CCI mouse model of TBI.

## Methods

### Animals and experimental design

These studies were approved by the University of Kentucky Institutional Animal Care and Usage Committee and the United States Veterans Affairs Animal Component of Research Protocol. Additionally, the Division of Laboratory Animal Resources at the University of Kentucky is accredited by the Association for the Assessment and Accreditation for Laboratory Animal Care International, and all experiments were performed with its guidelines. All animal experiments were compliant with ARRIVE (Animal Research: Reporting of In Vivo Experiments) guidelines, and experiments were carried out in accordance with the National Institutes of Health Guide for the Care and Use of Laboratory Animals.^37^ All experiments were conducted using adult male and female (2–3 months) wild-type C57BL/6 (The Jackson Laboratory, Bar Harbor, ME) mice with average masses of 25 (male) or 20 g (female). Animals were randomly assigned to either sham or CCI injury groups. Animals were housed 5 per cage and maintained in a 14-h light/10-h dark cycle, and all animals were housed in the same room. All animals were fed a balanced diet *ad libitum*, and water was reverse osmosis generated. The exact numbers of animals used in each experiment are reported in the figure legends, as well as the number of technical replicates utilized for each biological sample (if applicable).

### Mouse model of traumatic brain injury

Injury procedures were similar as previous published studies, with minor changes.^5,7^ Mice were initially anesthetized in a plexiglass chamber using 4.0% isoflurane and placed in a stereotaxic frame. During the injury procedure, anesthesia was maintained with 2.5% isoflurane delivered by a nose cone. The head was positioned in the horizontal plane with the nose bar set at zero. After an incision exposing the skull, a 4-mm craniotomy was made lateral to the sagittal suture and centered between the bregma and lambda. The skull cap at the craniotomy was carefully removed without damaging the underlying dura, and the exposed cortex was injured using a pneumatically controlled impactor device as described previously.^6^ The impactor tip diameter was 3 mm, and the impact velocity was set at 3.5 m/sec with a cortical depth of 1.0 mm. After injury, the craniotomy was closed by placement of a 4-mm sterile hemostatic dressing over the site. Animals were then placed in a recovery cage over a heating pad at 37°C until consciousness (i.e., return of righting reflex and mobility) was regained to prevent hypothermia. The sham group underwent anesthesia and craniotomy, but no cortical impact or injury to the dura mater.

### Isolation of mitochondria from brain tissue

Mice were rendered unconscious by carbon dioxide and immediately decapitated. Brains were removed, the ipsilateral hippocampi and 4-mm diameter cortical punches containing the area of injury were taken, and then tissues were homogenized in a Teflon-glass Dounce homogenizer in 1 mL of isolation buffer (215 mM of mannitol, 75 mM of sucrose, 0.1% bovine serum albumin [BSA], 20 mM of HEPES, and 1 mM of ethylene glycol tetraacetic acid, adjusted to a pH of 7.2 with KOH). Then, 50 μL of homogenate from each sample were snap-frozen on dry ice and reserved for future studies. The remaining homogenate was topped up to 2 mL with isolation buffer to continue with each respective isolation protocol at 4°C.

Total mitochondria were isolated from a punch of the injured cortex, as well as a punch from the contralateral cortex to serve as the uninjured control. Total mitochondria were isolated from these tissues 24 h post-injury by Ficoll density gradient ultracentrifugation (UC), as described previously, with modifications.^23,38^ Briefly, after a crude mitochondrial pellet was obtained as described in Vekaria and colleagues, pellets were resuspended in 500 μL of isolation buffer, and then the synaptic mitochondria were released from the synaptosomes using a pressurized nitrogen cell disruptor at 1200 psi for 10 min at 4⁰C.^38,39^ The disrupted samples were then placed onto the double-layer Ficoll gradient for UC.^23,38^

Synaptic and non-synaptic mitochondria were isolated using fractionated mitochondrial magnetic separation (FMMS), as previously described, for the time course of bioenergetics.^23^ This method of mitochondrial isolation has been shown to have a greater yield of synaptic mitochondria per milligram of tissue compared to UC, but the functionality of both synaptic and non-synaptic mitochondria isolated by FMMS is comparable to mitochondria isolated by UC.^23^ Synaptic and non-synaptic mitochondria were isolated 3, 12, 24, and 48 h post-CCI or sham surgery and used immediately for bioenergetic measurements.

For the calcium loading assay, a separate cohort of mice was euthanized by an overdose of sodium pentobarbital and then transcardially perfused with ice-cold calcium “locking” buffer (215 mM of mannitol, 75 mM of sucrose, 0.1% BSA, 20 mM of HEPES, 1.8 μM of ruthenium red, 10 μM of CGP-37157, and 5 μM of cyclosporin A, adjusted to a pH of 7.2 with KOH). This buffer contains a cocktail of inhibitors to inhibit the release of Ca^2+^ out of the mitochondrial matrix or any additional Ca^2+^ back into the matrix: ruthenium red to block the mitochondrial inward transport of Ca^2+^ by the uniporter and extrusion by the sodium-independent antiporter; CGP-37157 to block the outward flux of Ca^2+^ by the sodium-dependent antiporter; and cyclosporin A to prevent the loss of Ca^2+^ by induction of the permeability transition. Synaptic and non-synaptic mitochondria were isolated in locking buffer by Ficoll density gradient UC as described previously.^23,38^ This method of isolation was used for this assay because of the perceived confounding interaction between anti-TOM22 MicroBeads (Miltenyi Biotec Inc., Charlestown, MA) and CaG5N signal. These mitochondrial fractions were isolated 24 h post-CCI from the injured cortex, as well as the cortex contralateral to injury, to serve as the uninjured control to reduce animal use. To ensure that sufficient mitochondrial concentrations were obtained, each cortical sample was pooled from 2 mice.

Protein concentrations of all mitochondrial fractions were determined with the BCA Protein Assay kit (Cat # 23227; Pierce Biotechnology, Waltham, MA) by measuring absorbance at 560 nm on the Biotek Synergy HTX multi-mode plate reader (Agilent Technologies, Lexington, MA).

### Mitochondrial bioenergetic measurements

Immediately after isolation, mitochondrial bioenergetics were analyzed on the Seahorse XFe96 Analyzer (Agilent Technologies). Concentrations of mitochondria loaded per well are listed as follows: 1 ug total (Ficoll); 3 ug non-synaptic (FMMS), and 6 ug synaptic (FMMS). Oxygen consumption rates (OCRs) are measured in the presence of different substrates, inhibitors, and uncouplers of the mitochondrial electron transport chain (ETC) as described previously.^6^ State III respiration (respiration coupled to adenosine triphosphate [ATP] synthesis) is measured after the addition of pyruvate and malate (5 mM of pyruvate and 2.5 mM of malate; Port A), which are tricarboxylic acid (TCA) cycle substrates that feed to Complex I, and adenosine diphosphate (ADP; 4.3 mM), a necessary building block of ATP. State IV respiration (proton leak or respiration without ATP synthesis) is measured after the addition of oligomycin (2.5 μM; Port B), an inhibitor of ATP synthase. State V(CI) respiration (uncoupled respiration driven by Complex I) is measured after the addition of FCCP (4 μM; Port C), a mitochondrial uncoupler that carries protons back into the matrix, lowering ΔΨm. Finally, State V(CII) respiration (Complex II–driven maximum respiration) is measured after the addition of rotenone (0.8 μM), a Complex I inhibitor, and succinate (10 mM; Port D), a TCA cycle substrate that feeds into Complex II of the ETC. Each plate had three or more technical replicates per sample. Between experiments, plate-to-plate and day-to-day variability may result in variable OCR values, which necessitates the use of blocking factor in statistical analysis (Figs. 2–5).

### Calcium loading

Synaptic and non-synaptic mitochondrial calcium (Ca^2+^) capacity was assessed in a separate cohort of mice according to a previously published study that was modified for synaptic and non-synaptic mitochondria isolated by Ficoll density gradient UC.^9^ Ca^2+^ content of the mitochondria was assessed using the membrane-impermeable Ca^2+^ indicator, Calcium Green 5-N (catalog no.: C3737; ThermoFisherScientific, Waltham, MA), by measuring fluorescence (excitation, 506 + 10; emission, 532 + 10; gain 100) in intact mitochondria before and after the addition of the ionophore, alamethicin (catalog no.: A4665-10MG; Sigma-Aldrich, St. Louis, MO). A standard Ca^2+^ curve was generated (range, 3.125–150 μM) to extrapolate fluorescence values to concentrations of nanomoles of [Ca^2+^]/mg of mitochondrial protein. All assays and standards were performed using identical total volumes on the Cytation 5 multi-mode reader (Agilent Technologies). Each plate had two or more technical replicates per sample.

### Histology and tissue sparing

A separate cohort of mice was anesthetized by an overdose of sodium pentobarbital (95 mg/kg body weight) 7 days post-sham or post-CCI surgery. Mice were then transcardially perfused with ice-cold physiological saline followed by ice-cold buffered formalin (catalog no.: 89370-094; VWR International, Radnor, PA). Brains were extracted and post-fixed in formalin for 24 h. Brains were then transferred to a 30% sucrose in phosphate-buffered saline and stored for 2 weeks before sectioning. Then, 35-μm-thick coronal sections were cut using a freezing microtome beginning at the anterior commissure, continuing caudally through the entire area of injury, and ending at the most posterior extent of the hippocampus.^7^ Eight equally spaced sections were mounted and then stained using cresyl violet. Cortical lesion volume was quantified using the Calvalieri method, as described previously.^7,40,41^ In short, this method utilizes an unbiased stereological approach to quantify cortical damage across a systematic random subset of sections separated by a known distance (d = 420 μm). Sections were uploaded using the Axio Scan.Z1 (Carl Zeiss AG, Jena, Germany), and then the cortical areas ipsi- and contralateral to the injury were quantified using HALO imaging software (Indica Labs, Albuquerque, NM). Each area was multiplied by d to calculate the subvolume, and all of the subvolumes were summed to yield the estimated total volume. Tissue spared on the ipsilateral cortex was calculated as a percentage of the contralateral cortex: [(volume of ipsilateral cortex) / (volume of contralateral cortex) × 100].

### Statistical analysis

Bioenergetic experiments were designed for an *a priori* analysis of males versus females. Power analysis was conducted using G*Power software (v.3.1.9.7), using the following assumptions: α = 0.05, 1 – β = 0.8, and standard deviation = 10% (mitochondrial outcomes) or 25% (histological outcomes) of the mean.^42^ All statistical analyses were performed in GraphPad Prism (GraphPad Software Inc., La Jolla, CA) or JMP Pro 16 (SAS Institute Inc., Cary, NC). Brown-Forsythe and Bartlett's tests were used to ensure homogeneity of variance, and the Shapiro-Wilk test was used to assess normality. Because all data passed the normality assumptions, parametric tests were used for all analyses. All mitochondrial experiments were analyzed by two-way analysis of variance (ANOVA) with Sidak's multiple comparisons test, where applicable, with sex and injury as residuals. Raw OCR values were converted to the percentage of sham (100%) for the respective sex on that particular Seahorse plate. These values were compared to sham by a one-tailed *t*-test. Outliers of technical replicates were identified using the interquartile rule: Any value greater than the upper limit (1.5 × interquartile range [IQR] + third quartile) or less than the lower limit (first quartile – 1.5 × IQR) was considered an outlier and subsequently removed from the data set. To account for plate-to-plate and day-to-day variability in absolute OCR values, we used a blocking factor of the assay day (i.e., Seahorse plate) when performing the two-way ANOVA.

## Results

### Synaptic and non-synaptic mitochondrial bioenergetics

It is well established in experimental TBI models in male rodents that there is a significant bioenergetic impairment in total cortical mitochondria 24 h after CCI.^5,6,12^ Additionally, Greco and colleagues have shown that male rats had significant impairments in the mitochondrial respiratory control ratio 24 h post-CCI, whereas total mitochondria from females did not show this injury effect.^36^ We observed a significant injury effect in State III and State V(CII) respiration in total mitochondria from the injured cortex of male mice 24 h post-CCI compared to the contralateral cortical control; however, this injury effect was not present in females (Supplementary Fig. S1). To delve deeper into this initial observed sex difference, we measured cortical and hippocampal mitochondrial bioenergetics at different time points post-injury in synaptic and non-synaptic mitochondrial fractions.

#### Three hours

**FIG. 1.**
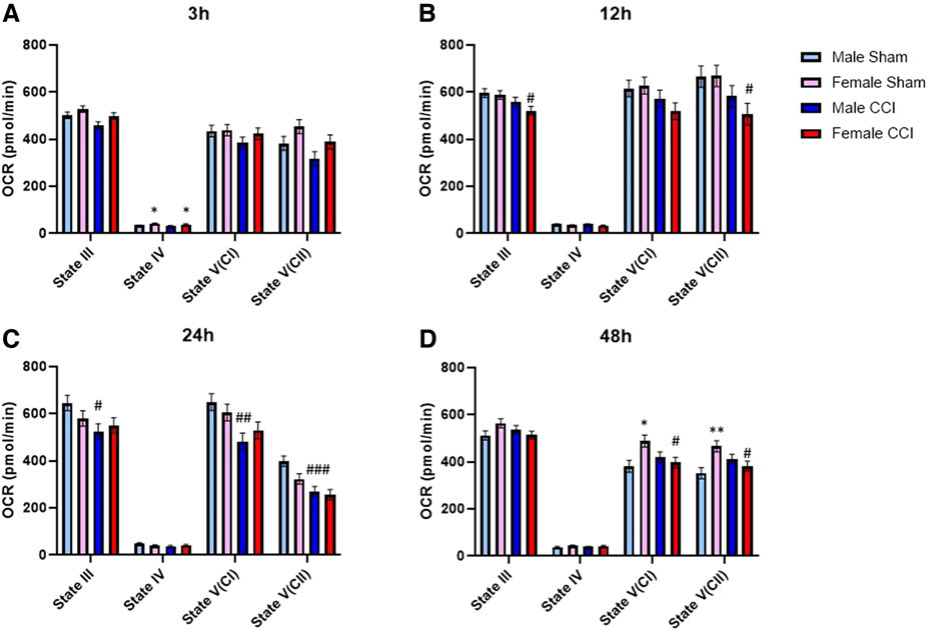
Non-synaptic mitochondria from the cortex of females showed earlier bioenergetic dysfunction compared to males. State III respiration is measured after the addition of pyruvate, malate, and ADP to give ATP-production-linked respiration. State IV respiration is measured after the addition of oligomycin to give respiration driven by proton leak. State V(CI) respiration is measured after the addition of FCCP, a mitochondrial uncoupler that carries protons back into the matrix, to give uncoupled respiration driven by complex I. State V(CII) respiration is measured after the addition of rotenone (complex I inhibitor) and succinate, to give uncoupled respiration driven by complex II. To account for plate-to-plate and day-to-day variability, a blocking factor was used. First, 3 μg of non-synaptic mitochondria were loaded per well. Male/Female Sham = craniotomy without impact; Male/Female CCI = craniotomy with impact. Values are represented as mean ± SEM; *n* = 5–6 mice with three or more technical replicates. Each state was analyzed by two-way ANOVA with Sidak's multiple comparisons, where appropriate. Compared to male of respective injury group: *p < 0.05; **p < 0.01. Compared to shams of respective sex: ^#^*p* < 0.05; ^##^*p* < 0.01; ^###^*p* < 0.001. ADP, adenosine diphosphate; ANOVA, analysis of variance; ATP, adenosine triphosphate; CCI, controlled cortical impact; OCR, oxygen consumption rate; SEM, standard error of the mean.

**FIG. 2.**
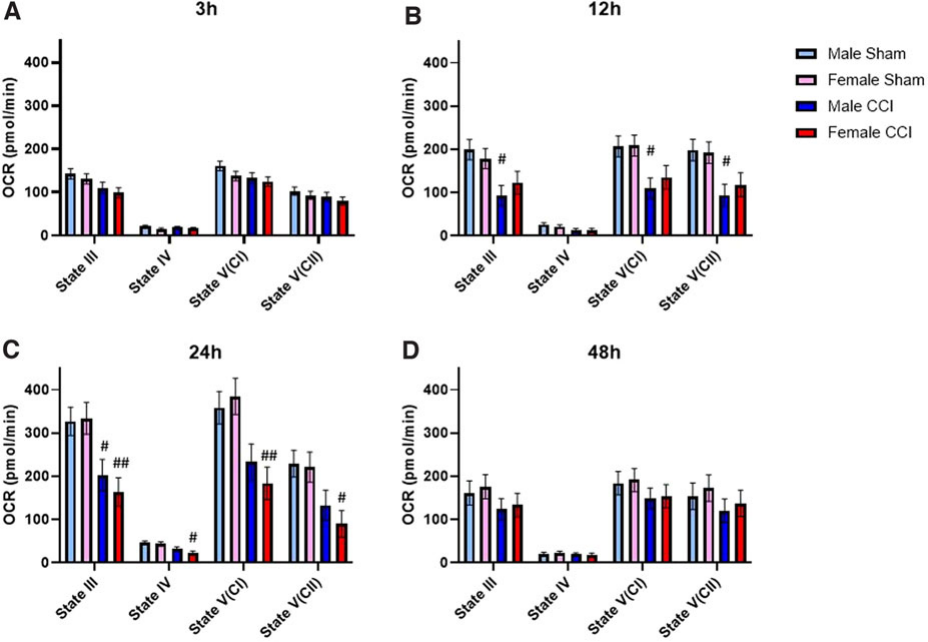
Synaptic mitochondria from the cortex of males showed earlier bioenergetic dysfunction compared to females. State III respiration is measured after the addition of pyruvate, malate, and ADP to give ATP-production-linked respiration. State IV respiration is measured after the addition of oligomycin to give respiration driven by proton leak. State V(CI) respiration is measured after the addition of FCCP, a mitochondrial uncoupler that carries protons back into the matrix, to give uncoupled respiration driven by complex I. State V(CII) respiration is measured after the addition of rotenone (complex I inhibitor) and succinate, to give uncoupled respiration driven by complex II. To account for plate-to-plate and day-to-day variability, a blocking factor was used. Male/Female Sham = craniotomy without impact; Male/Female CCI = craniotomy with impact. First, 6 μg of synaptic mitochondria were loaded per well. Values are represented as mean ± SEM; *n* = 5–6 mice with two or more technical replicates. Each state was analyzed by two-way ANOVA with Sidak's multiple comparisons, where appropriate. Compared to sham of respective sex: ^#^*p* < 0.05; ^##^*p* < 0.01. ADP, adenosine diphosphate; ANOVA, analysis of variance; ATP, adenosine triphosphate; CCI, controlled cortical impact; OCR, oxygen consumption rate; SEM, standard error of the mean.

**FIG. 3.**
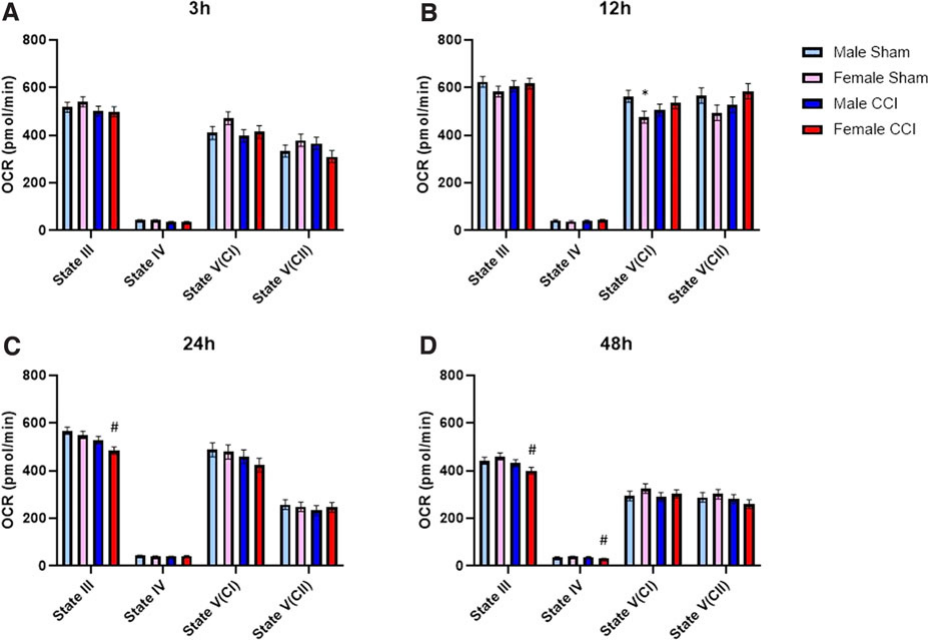
Non-synaptic mitochondria from the hippocampus of females showed delayed bioenergetic impairment, whereas mitochondria from males showed no impairment across time. State III respiration is measured after the addition of pyruvate, malate, and ADP to give ATP-production-linked respiration. State IV respiration is measured after the addition of oligomycin to give respiration driven by proton leak. State V(CI) respiration is measured after the addition of FCCP, a mitochondrial uncoupler that carries protons back into the matrix, to give uncoupled respiration driven by complex I. State V(CII) respiration is measured after the addition of rotenone (complex I inhibitor) and succinate, to give uncoupled respiration driven by complex II. To account for plate-to-plate and day-to-day variability, a blocking factor was used. First, 3 μg of non-synaptic mitochondria were loaded per well. Male/Female Sham = craniotomy without impact; Male/Female CCI = craniotomy with impact. Values are represented as mean ± SEM; *n* = 5–6 mice with three or more technical replicates. Each state was analyzed by two-way ANOVA with Sidak's multiple comparisons, where appropriate. Compared to male of respective injury group: *p < 0.05. Compared to sham of respective sex: ^#^*p* < 0.05. ADP, adenosine diphosphate; ANOVA, analysis of variance; ATP, adenosine triphosphate; CCI, controlled cortical impact; OCR, oxygen consumption rate; SEM, standard error of the mean.

**FIG. 4.**
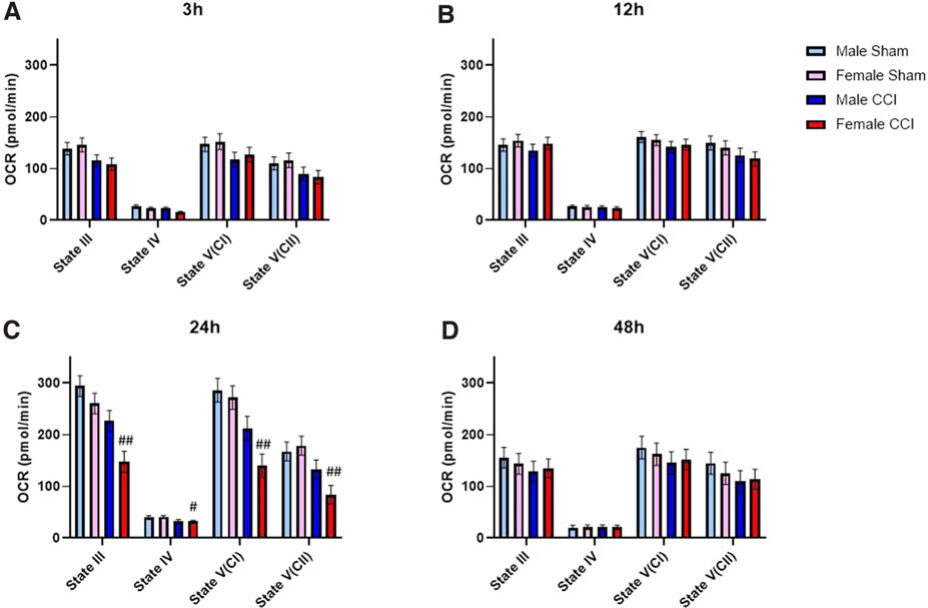
Synaptic mitochondria from the hippocampus of females only showed bioenergetic impairment at 24 h post-CCI, whereas mitochondria from males showed no impairment across time. State III respiration is measured after the addition of pyruvate, malate, and ADP to give ATP-production-linked respiration. State IV respiration is measured after the addition of oligomycin to give respiration driven by proton leak. State V(CI) respiration is measured after the addition of FCCP, a mitochondrial uncoupler that carries protons back into the matrix, to give uncoupled respiration driven by complex I. State V(CII) respiration is measured after the addition of rotenone (complex I inhibitor) and succinate, to give uncoupled respiration driven by complex II. To account for plate-to-plate and day-to-day variability, a blocking factor was used. First, 6 μg of synaptic mitochondria were loaded per well. Male/Female Sham = craniotomy without impact; Male/Female CCI = craniotomy with impact. Values are represented as mean ± SEM; *n* = 5–6 mice with three or more technical replicates. Each state was analyzed by two-way ANOVA with Sidak's multiple comparisons, where appropriate. Compared to sham of respective sex: ^#^*p* < 0.05; ^##^*p* < 0.01. ANOVA, analysis of variance; CCI, controlled cortical impact; OCR, oxygen consumption rate; SEM, standard error of the mean.

Results showed that there was a difference in the main effect for both sex and injury for both State III and State V(CII) respiration from non-synaptic cortical mitochondria; however, there were no statistically significant differences between groups (Fig. 1A). We did observe increased State IV respiration in non-synaptic mitochondria from both sham- and CCI-injured females compared to the respective male groups (Fig. 1A). In synaptic cortical mitochondrial fractions, there were no significant differences between sex or injury group at any respiration state (Fig. 2A). There were no observed sex differences nor injury effects in either non-synaptic or synaptic hippocampal mitochondrial fraction at any respiration state (Figs. 3A and 4A).

#### Twelve hours

At 12 h post-injury, we found that non-synaptic mitochondria from the injured cortex of females had significantly impaired State III and State V(CII) respiration compared to sham females (Fig. 1B). Non-synaptic cortical mitochondria from male mice did not have a significant injury effect compared to male sham (Fig. 1B). However, synaptic mitochondria from the injured cortex of male mice had significant bioenergetic impairment across all respiration states, excluding State IV, compared to male sham (Fig. 2B). This injury effect was not statistically significant in synaptic cortical mitochondria from females (Fig. 2B). State V(CI) respiration of sham female-derived hippocampal mitochondria was significantly lower than that of sham males (Fig. 3B). There were no other observed sex differences nor injury effects in either non-synaptic or synaptic hippocampal mitochondrial fraction at any respiration state (Figs. 3B and 4B).

#### Twenty-four hours

Non-synaptic cortical mitochondria from CCI-injured male mice were significantly impaired across State III, State V(CI), and State V(CII) respiration compared to sham male; however, this effect was not observed in non-synaptic mitochondria from females (Fig. 1C). Synaptic mitochondria from the injured cortex of male mice only had statistically significant bioenergetic deficits in State III respiration compared to male sham (Fig. 2C). Synaptic mitochondria from the cortex of injured female mice showed bioenergetic deficits across all respiration states compared to female sham (Fig. 2C). Non-synaptic hippocampal mitochondria from CCI-injured females had significantly lower State III respiration compared to that of sham females (Fig. 3C). Synaptic hippocampal mitochondria from injured female mice was significantly impaired across all respiration states compared to their sham control (Fig. 4C). There were no observed injury effects across any respiration states between the male groups, nor were there any detected sex differences in either hippocampal mitochondrial fraction (Figs. 3C and 4C).

#### Forety-eight hours

Non-synaptic cortical mitochondria from male mice did not show an injury effect across any respiration states (Fig. 1D). Bioenergetics of non-synaptic mitochondria from the cortex of injured females were significantly impaired across State V(CI) and State V(CII) respiration compared to female sham (Fig. 2D). Additionally, there was an observed sex difference between the sham groups at State V(CI) and State V(CII) respiration, in which mitochondria from females had significantly higher respiration than mitochondria from males (Fig. 2D). Non-synaptic hippocampal mitochondria from injured female mice had significantly impaired State III and State IV respiration compared to that of sham female (Fig. 3D). There were no observed differences in synaptic hippocampal mitochondrial respiration at any state with respect to sex and injury group (Fig. 4D).

**FIG. 5.**
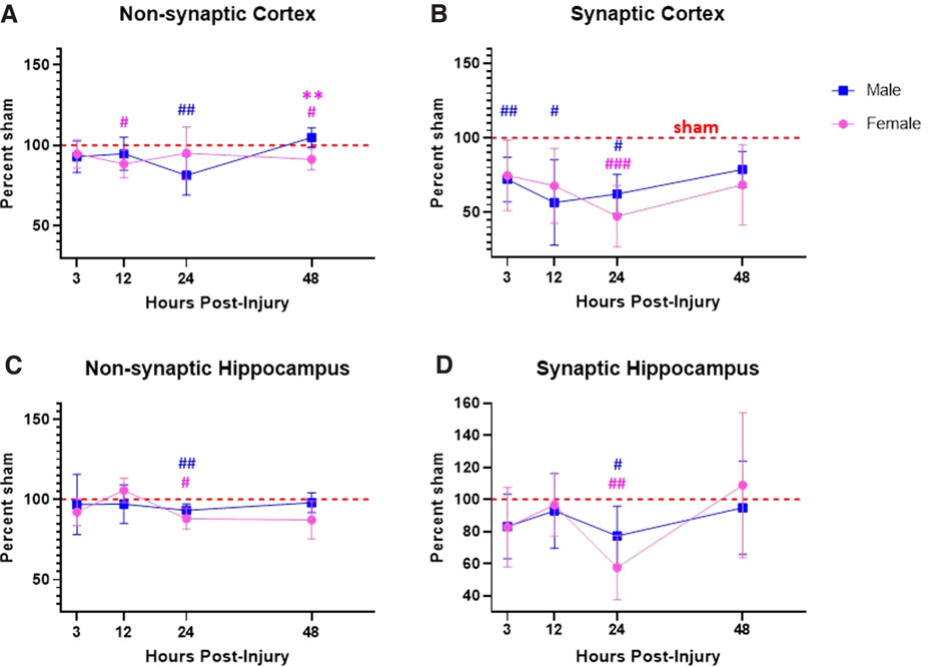
Time course of State III respiration between the sexes in non-synaptic and synaptic mitochondria from the cortex and hippocampus of male and female mice. Raw OCR values were converted to percent of sham (100%) within Seahorse plates for each sex. Values are represented as mean ± SD; *n* = 5–6 mice. Each time point was analyzed by a one-tailed unpaired *t*-test. Compared to males of respective injury group: **p < 0.01. Compared to the sham of respective sex: ^#^*p* < 0.05; ^##^*p* < 0.01, ^###^*p* < 0.001. OCR, oxygen consumption rate; SD, standard deviation.

State III respiration, as a percentage of sham in synaptic and non-synaptic mitochondria from the injured cortex and hippocampus across time, is summarized in Figure 5.

### Synaptic and non-synaptic mitochondrial calcium loading

**FIG. 6.**
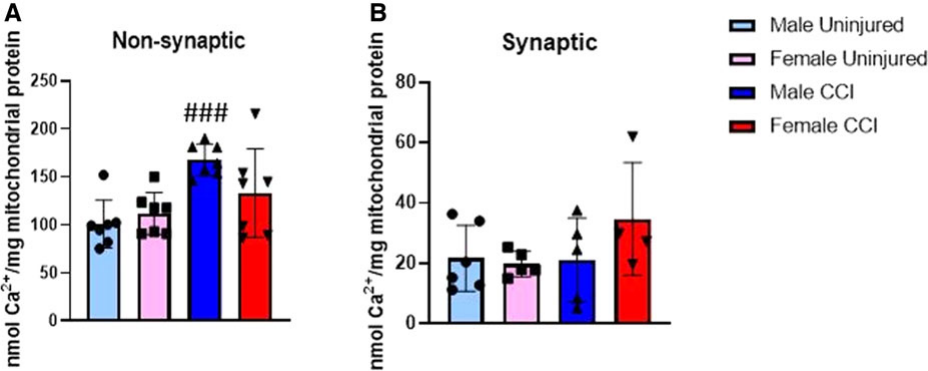
Non-synaptic mitochondrial calcium loading was increased 24 h post-CCI in males, but not in females. Non-synaptic (**A**) and synaptic (**B**) cortical mitochondrial calcium loading 24 h post-CCI in male and female mice. Male/Female Uninjured = cortex contralateral to injury; Male/Female CCI = cortex ipsilateral to injury. Values are represented as mean ± SD; *n* = 4–7 mice with two or more technical replicates. Analyzed by two-way ANOVA with Sidak's multiple comparisons, where appropriate. Compared to sham or respective sex: ^###^*p* < 0.001. ANOVA, analysis of variance; CCI, controlled cortical impact; SD, standard deviation.

To further characterize sex differences in mitochondrial function, we observed calcium loading in non-synaptic and synaptic mitochondria 24 h post-CCI, the time in which we observed maximum bioenergetic deficits in synaptic, but not non-synaptic, mitochondria from cortices of female mice. We observed increased calcium loading in non-synaptic mitochondria from the ipsilateral CCI-injured cortex in males compared to the uninjured contralateral cortex (Fig. 6A). However, this effect was not observed in synaptic mitochondria from males (Fig. 6B). There were no significant increases in calcium loading in either ipsilateral mitochondrial fractions after injury in females compared to their respective contralateral controls (Fig. 6A,B).

### Tissue sparing assessment

**FIG. 7.**
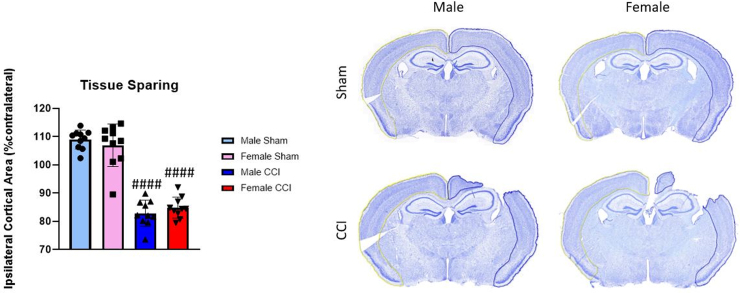
There was a lack of sex differences in cortical tissue sparing at 7 days post-CCI. Mice were euthanized, and the brains were fixed 7 days post-injury. Tissue spared on the ipsilateral cortex (blue outline) was calculated as a percent of the contralateral cortex (yellow outline): [(volume of ipsilateral cortex) / (volume of contralateral cortex) × 100]. Male/Female Sham = craniotomy without impact; Male/Female CCI = craniotomy with impact. Values are represented as mean ± SD; *n* = 10 mice per group. Each measurement was analyzed by two-way ANOVA with Sidak's multiple comparisons. Compared to sham of respective sex: ^####^*p* < 0.0001. ANOVA, analysis of variance; CCI, controlled cortical impact; SD, standard deviation.

It is well established that the CCI model of TBI produces a significant cortical lesion. The present literature has conflicting results regarding sex differences in lesion volume in adult mice after moderate-severe CCI, in which there are either no sex differences or smaller lesion volumes in females.^43–48^ Here, we measured cortical tissue sparing in adult male and female mice 7 days after severe CCI (Fig. 7). The results showed extensive cortical damage in both sexes post-CCI compared to their respective shams; however, there were no sex differences detected in spared cortical tissue.

## Discussion

TBI is a heterogeneous condition, which is represented by all of the confounding data available in both humans and animals.^10,49^ Severe TBI was selected for these studies based on the review by Gupte and colleagues, which found that a large percentage of both human and animal studies reported better outcomes in females compared to their male counterparts after severe TBI.^10^ These experiments were aimed to identify whether mitochondria could play a role in these apparent sex differences after severe CCI in mice. Additionally, it has been shown in total mitochondria from rats that there are sex differences in the respiratory control ratio 24 h after severe CCI.^36^ No other studies to date have examined whether there are sex differences in synaptic and non-synaptic mitochondrial function after any degree of TBI severity. The results from these studies highlight the importance of measuring synaptic and non-synaptic mitochondrial function, rather than just total brain mitochondrial function. Additionally, these studies highlight the importance of observing both sexes for mitochondrial outcomes. The results have been summarized in Table 1.

**Table 1.** Experimental Results Summary

Bioenergetics	Male	Female	Sex difference
Non-synaptic cortex	Impairments began at 24 h; resolved by 48 h	Impairments began at 12 h; restored by 24 h; impaired at 48 h	3 h: ^*^ 12 h: ns 24 h: ns 48 h: ns
Synaptic cortex	Impairments began at 12 h; resolved by 48 h	Impairments began at 24 h; resolved by 48 h	3 h: ns 12 h: ns 24 h: ns 48 h: ns
Non-synaptic hippocampus	No impairments	Impairments began 24 h; persisted at 48 h	3 h: ns 12 h: ns 24 h: ns 48 h: ns
Synaptic hippocampus	No impairments	Impairments began at 24 h; resolved by 48 h	3 h: ns 12 h: ns 24 h: ns 48 h: ns

Calcium loading	Male	Female	Sex difference
Non-synaptic cortex	Increased Ca^2+^ load at 24 h	No impairments	ns
Synaptic cortex	No impairments	No impairments	ns

Cortical tissue sparing	Male	Female	Sex difference
Percent of contralateral cortex	83% tissue spared	85% tissue spared	ns

^*^ Sex difference = statistically significant (*p* < 0.05) main sex effect. For this summary of bioenergetics, sex difference was identified as showing the significant main sex effect for at least two out of four respiration states for that given time point.

ns, not significant

Total mitochondria from the cortex of male mice had significantly impaired State III and State V(CII) respiration compared to male sham, but mitochondria from females did not have this injury effect 24 h post-injury (Supplementary Fig. S1). When looking at both mitochondrial fractions, we found that both synaptic and non-synaptic mitochondria from male mice were significantly impaired 24 h post-injury. Synaptic mitochondria from female mice were significantly impaired 24 h post-injury (Fig. 2C); however, non-synaptic mitochondrial bioenergetic function was spared (Fig. 1C). This indicates that synaptic mitochondrial dysfunction was masked by spared non-synaptic function in the total mitochondrial population in female mice.^24^ Based on these data, studies should be powered to detect sex differences, especially when examining synaptic and non-synaptic mitochondria, so that spared function in one sex or mitochondrial fraction does not mask impairments in others.

The only statistically significant sex differences in the time course of bioenergetics were found in the non-synaptic mitochondrial fractions from both the cortex and hippocampus. At 3 h, non-synaptic cortical mitochondria from both sham- and CCI-injured females had increased State IV respiration compared to their respective male injury group (Fig. 1A). At 12 h, non-synaptic mitochondria from the hippocampus of sham females had lower State V(CI) respiration compared to male sham (Fig. 3B). Finally, at 48 h, non-synaptic mitochondria from the cortex of sham females had increased State III and V(CI) respiration at 48 h compared to male sham (Fig. 1D). Besides those aforementioned sex differences, there were no other statistically significant variations between sexes at any respiration state or time point in either tissue or mitochondrial fraction. However, there were differences in the time course of mitochondrial dysfunction post-CCI, in which one sex showed bioenergetic impairment whereas the other did not. Hill and colleagues have shown that synaptic mitochondria undergo bioenergetic dysfunction before non-synaptic mitochondria in male rats.^12^ These present studies observed a similar pattern of early cortical synaptic mitochondrial impairment at 12 h post-CCI (Fig. 2B), followed by non-synaptic mitochondrial dysfunction at 24 h post-CCI (Fig. 1C).

Mitochondria from the cortex of female mice revealed a different pattern of injury. Beginning at 12 h post-CCI, synaptic and non-synaptic mitochondria appeared to alternate dysfunction, such that at 12 h non-synaptic mitochondria were impaired (Fig. 1B) and synaptic mitochondria were not (Fig. 2B). Then, at 24 h post-injury, non-synaptic mitochondrial function was spared (Fig. 1C), but synaptic mitochondrial function was impaired (Fig. 2C). Then, at 48 h, mitochondrial dysfunction switched again: Non-synaptic mitochondria were impaired (Fig. 1D) and synaptic mitochondria were spared (Fig. 2D). These results are easily visualized when the data are converted to percentage of sham across time (Fig. 5). It is clear to see that cortical mitochondria from males follows the canonical pattern of early synaptic dysfunction, followed by delayed non-synaptic dysfunction (Fig. 5A,B). Then, in females, we observe the alternation of synaptic and non-synaptic mitochondrial impairment across time (Fig. 5A,B). In the hippocampus, we observe non-synaptic and synaptic mitochondrial impairment in both sexes 24 h post-CCI (Fig. 5C,D). These results indicate that time post-injury, mitochondrial fraction, and brain region should be carefully considered when planning experiments to include sex as a biological variable.

Excitotoxicity after TBI causes an increase in intramitochondrial Ca^2+^ content.^8,9^ These present studies also found increased Ca^2+^ content in non-synaptic mitochondria from the cortex of male mice 24 h post-CCI, though this effect was not observe in females (Fig. 6A). This is consistent with our bioenergetic data at that time point, which showed that cortical non-synaptic mitochondria from male mice were significantly impaired, whereas the non-synaptic mitochondria from females were not (Fig. 1C). These data continue to drive home the importance of using both sexes when measuring mitochondrial dysfunction after TBI so that injury effects in one sex are not masked by spared function in another. Synaptic mitochondrial Ca^2+^ content was not significantly impaired in either sex after severe CCI (Fig. 6B), though the results were trending up in the injured female group to be consistent with bioenergetic data (Fig. 2C). Retrospective power analysis determined that a sample size of 19 mice per group would be necessary to observe a significant injury effect between the female groups. The lack of an injury effect in this fraction could be attributable to the sensitivity of synaptic mitochondria to TBI- and Ca^2+^-induced damage. It is possible that the synaptic mitochondria have already undergone permeability transition and released Ca^2+^ before they were locked by the isolation buffer, resulting in Ca^2+^ concentrations similar to the uninjured cortex.^12,18,19^ However, these mitochondria would still be bioenergetically impaired, given that permeability transition collapses mitochondrial membrane potential and uncouples oxidative phosphorylation.^3,50^

Several studies have measured sex differences in cortical lesion volume after moderate-severe CCI and have conflicting results.^43–48^ Studies by Tucker and colleagues and Jullienne and colleagues have reported no sex differences in lesion volume, whereas Clevenger and colleagues, Igarashi and colleagues, and Villapol and colleagues reported smaller lesion volume in females compared to males. Tucker and colleagues utilized a 1-mm-depth CCI, but classified it as a mild injury, which is consistent with their analysis showing that lesion volumes in both sexes were not significantly different from their respective sham groups 30 days post-CCI.^43^ We classify our 1-mm-depth contusion model of injury as severe because it mimics the following human pathologies: blood–brain barrier disruption, significant cortical tissue loss, and significant loss of hippocampal neurons, as described previously.^40,51-54^ We did not identify any differences in tissue sparing between the sexes, but both CCI groups were significantly lower than their respective sham groups 7 days post-CCI (Fig. 7). A study by Clevenger and colleagues measured cortical lesion volume 7 days after severe (2-mm-depth) CCI in male, intact female, and OVX female mice, and found that intact female mice had a smaller lesion volume compared to both male and OVX female mice.^45^ Another study found that female mice had a smaller lesion volume than males both 3 and 7 days after 1.5-mm-depth CCI; however, this injury effect was lost 30 days post-injury.^46^ Together, these studies reveal sex differences in lesion volume after a 1.5- to 2.0-mm-depth CCI, whereas these effects were not observed with ≤1-mm-depth CCI or chronically after injury.

Based on the terminology from Arambula and colleagues, the acute sex differences we observed in mitochondrial dysfunction after 1-mm-depth CCI converge on the same histological outcome.^55^ At this point, it is unclear whether there would be a divergence in functional outcomes after severe CCI between the sexes. Based on the aforementioned review by Gupte and colleagues, we could infer that females would have improved functional outcomes after severe CCI compared to males, though this requires further investigation.^10^ Additionally, whether the hypothesized sex differences in functional outcomes correlate with acute mitochondrial sex differences is a future question that needs to be answered.

We observed some deficits in hippocampal synaptic and non-synaptic mitochondrial bioenergetics in female mice, but not males. This could suggest that females would have more cognitive deficits compared to males, but this was not explored further in this study. Our lab has shown the correlation between synaptic mitochondrial function and cognitive outcomes in male mice, though this avenue should be explored further to determine whether this is true in both sexes.^6,24^ Estrus cycle phase was determined on the day of mitochondrial isolation, but not at the time of impact. As a result, there were not sufficient numbers of mice in each phase, for each time point, for each day of experimentation to perform proper statistical analyses on the effects of estrus cycle phase on mitochondrial function. This variation would make the female population in this study consistent with the clinical population given that it is impossible to control when a TBI happens; however, it is still a limitation to this study. Gaignard and colleagues have shown that brain mitochondrial respiration was not different between each phase of the estrus cycle, though this was determined in naïve, uninjured female mice.^31^ A study by Robertson and colleagues found that progesterone replacement in OVX female rats improved mitochondrial bioenergetics 1 h post-CCI.^56^ No other studies have observed the effect of estrogen replacement on mitochondrial function post-TBI, but results from naïve rats found that estrogen replacement improved brain mitochondrial bioenergetics compared to OVX control.^57^

A study recently found that men had worsened, but not statistically significant, cerebral energy metabolism likely attributable to mitochondrial dysfunction after severe TBI compared to women.^58^ Though these findings are notable, the researchers did not have access to the hormonal status of each patient so it is not known how or if these results correlate to hormone levels in either sex. With regard to the experiments conducted in this article, future studies will examine the role of estrogen on mitochondrial dysfunction after CCI in mice.

In conclusion, the lack of injury effect in total mitochondrial bioenergetics 24 h post-injury in females (Supplementary Fig. S1) prompted further investigation of the time course of synaptic and non-synaptic mitochondrial bioenergetic dysfunction. We did not find statistically significant sex differences in bioenergetics, though there were variations in the time course of injury. Mitochondria from males followed the more canonical pattern of early synaptic dysfunction (12 h), followed by delayed non-synaptic dysfunction (24 h). Compared to males, mitochondria from females showed an altered time course of dysfunction between mitochondrial fractions. Non-synaptic mitochondria from males had increased calcium loading 24 h post-injury compared to male sham. This is consistent with non-synaptic bioenergetic impairment observed in males at this time point. Conversely, synaptic mitochondria from either sex did not show an injury effect in calcium loading, even though both showed bioenergetic impairment at 24 h post-injury. There were no sex differences in cortical tissue sparing, though there were significantly less cortical tissue spared in both males and females 7 days post-CCI. Overall, the results presented in this article further drive home the importance of measuring mitochondrial dysfunction in both synaptic and non-synaptic fractions rather than just total mitochondria. Additionally, this study highlights the importance of powering experiments to identify sex differences, especially when assessing mitochondrial dysfunction after TBI, so that results are not masked by spared function in one sex over dysfunction in the other.

## Supplementary Material

Supplemental data

## Abbreviations Used

ADP: adenosine monophosphate
ATP: adenosine triphosphate
BSA: bovine serum albumin
CCI: controlled cortical impact
ETC: electron transport chain
FMMS: fractionated mitochondrial magnetic separation
IQR: interquartile range
OCR: oxygen consumption rate
RNS: reactive nitrogen species
ROS: reactive oxygen species
TBI: traumatic brain injury
TCA: tricarboxylic acid
UC: ultracentrifugation
