# Supplementary material for: Characterizing Sex Differences in Mitochondrial Dysfunction After Severe Traumatic Brain Injury in Mice
Source: Neurotrauma Rep. 2023 Sep 25;4(1):627–42. doi: 10.1089/neur.2023.0046 (PMC10518693; doi:10.1089/neur.2023.0046)

**Supplemental Figure Legends**

**Supplemental Figure 1.** **Total mitochondria from injured males, but not females, showed bioenergetic impairment 24h post-CCI.** State III respiration is measured after the addition of pyruvate, malate, and ADP to give ATP-production-linked respiration. State IV respiration is measured after the addition of oligomycin to give respiration driven by proton leak. State V(CI) respiration is measured after the addition of FCCP, a mitochondrial uncoupler that carries protons back into the matrix to give uncoupled respiration driven by complex I. State V(CII) respiration is measured after the addition of rotenone (complex I inhibitor) and succinate, to give uncoupled respiration driven by complex II. 1µg total mitochondria were loaded/well. Male/Female Uninjured = cortex contralateral to injury; Male/Female CCI = cortex ipsilateral to injury. Values are represented as mean ± SD; n=4 mice with ≥ 3 technical replicates. Values were analyzed by two-way ANOVA with Sidak’s multiple comparisons, where appropriate. Compared to male control: *p < 0.05; **p < 0.01.


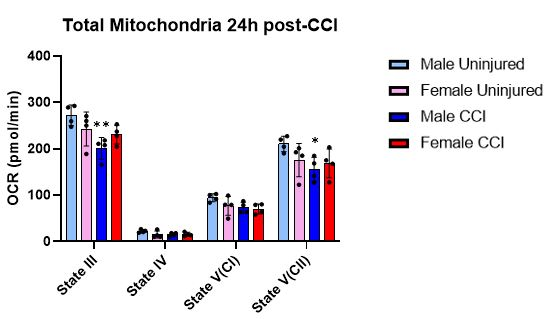

Supplement: Supplemental data [file Suppl_FigS1.docx]
